# Social support and subjective burden in caregivers of adults and older adults: A meta-analysis

**DOI:** 10.1371/journal.pone.0189874

**Published:** 2018-01-02

**Authors:** Rafael del-Pino-Casado, Antonio Frías-Osuna, Pedro A. Palomino-Moral, María Ruzafa-Martínez, Antonio J. Ramos-Morcillo

**Affiliations:** 1 Department of Nursing, Faculty of Health Sciences, University of Jaén, Jaén, Spain; 2 Department of Nursing, Faculty of Nursing, University of Murcia, Murcia, Spain; University of Illinois at Chicago College of Medicine, UNITED STATES

## Abstract

**Background:**

Despite the generally accepted belief that social support improves caregiver adjustment in general and subjective burden in particular, the literature shows mixed findings, and a recent review concluded that the predictive strength of caregiver social support in determining caregiver burden is less evident, due to the conceptual diversity of this determinant.

**Objective:**

The purpose of this review is to analyse the relationship of perceived and received social support with subjective burden among informal caregivers of an adult or older adult.

**Methods:**

A systematic search was carried out up to September 2017 in the following databases: MEDLINE (PubMed), CINAHL, EMBASE, PsycINFO), Scopus and ISI Proceedings, and a meta-analysis was performed with the results of the selected and included studies.

**Results:**

Fifty-six studies were included in the meta-analysis, which provided 46 independent comparisons for perceived support and 16 for received support. Most of these studies were cross-sectional. There was a moderate, negative association of perceived social support on subjective burden (*r* = -0.36; CI 95% = -0.40, -0.32) and a very small, negative association of received support on subjective burden (*r* = -0.05; CI 95% = -0.095, -0.001).

**Conclusions:**

1) perceived and received support are not redundant constructs, 2) the relationships between social support and subjective burden depend on whether the social support is measured as perceived or received, 3) the relationship of perceived social support with subjective burden has a bigger effect size than that of received social support, the relation between received support and subjective burden being clinically irrelevant, 4) perceived social support may be a good predictor of subjective burden.

**Implications of key findings:**

Our findings broadly support interventions promoting social support in caregivers to prevent or alleviate subjective burden, and specifically, to intervene on the promotion of perceived social support more than on the promotion of received social support when preventing or alleviating burden.

## Introduction

Social support can be defined as “the existence or availability of people on whom we can rely, people who let us know that they care about, value and love us” [1]. Classically, this construct has been classified in two dimensions: structural and functional. The structural dimension refers to the size, composition and complexity of the social network [2,3]. The functional dimension comprises the functional types of assistance (given or available) which can usually be classified into: emotional, instrumental and informational [4]. In addition, the functional dimension can be measured in two ways: perceived and received [5,6]. Perceived support refers to the assessment of the availability of support when it is needed, the appraisal of its adequacy and/or the quality of such support, whereas received support refers to the nature and frequency of specific support transactions [5,6].

Several researchers [7,8] have shown the positive effects of social support in psychological adjustment, health and well-being during the past three decades. However, despite these findings, limited progress has been made in understanding how social support works and in the specific mechanism linking social support and their benefits [6].

### Social support and caregiving

Social support has been studied in several contexts, one of which is informal caregiving. The study of family caregiving is a good opportunity to analyse how social support is related to psychological outcomes [9]. Caring for a relative is a stressful event that can have negative effects on caregivers’ health and well-being [10]. In caregiving, social support has been analysed under the stress and coping models derived from the Transactional Stress Theory by Lazarus and Folkman [11]. In these models, the consequences of the potential stressful events depend on the caregiver’s personal appraisal of these events and the caregiver’s resources such as social support. Based on these models, some authors have tried to theorize how social support modulates the stress consequences. In this sense, Cohen et al. [12,13] argued that social support may play a role at two different points in the causal sequence, linking stress to its consequences. First, the perception that others can provide necessary resources could lead to appraising a situation as less stressful. Second, the actual receipt of support may alleviate the impact of stress by providing a solution to the problem, by reducing the perceived importance of the problem, by providing distraction from the problem or by facilitating healthful behaviours. Thus, social support could diminish the impact of stressors on caregiver’s emotional situation [12,13].

### Social support and subjective burden

Our study is focused on the relationship between social support and subjective caregiver burden. Subjective burden is a state characterized by fatigue, stress, perceived limited social contact and role adjustment, and perceived altered self-esteem. This state comes from a negative appraisal of the caregiving situation, and can threaten the physical, psychological, emotional and functional health of caregivers [14,15]. Subjective burden has been related to anxiety [16], depression [17,18], and negative effects on physical health [19]. Thus, the analysis of how social support affects subjective burden could improve the health of the caregiver through early detection and early interventions on subjective burden.

Despite the generally accepted belief [9] that social support improves caregiver adjustment in general and subjective burden in particular, the literature shows mixed findings [9,20], existing works have linked social support to less subjective burden [21], more subjective burden [22] or shown no relationship [23]. Moreover, a recent review about social functioning (including social support) and subjective burden in dementia caregivers [24] concluded that the predictive strength of caregiver social support in determining caregiver burden is less evident, due to the conceptual diversity of this determinant.

Researchers have tried to explain previous heterogeneity and scarcity of evidence by analysing perceived and received social support separately, based on the hypothesis that perceived support has more consistently related to beneficial health outcomes than received social support [25]. However, results in the last review about the relationships between social support and psychological adjustment in caregivers (including subjective burden) [9] were not conclusive because of the heterogeneity of its findings, the few studies included and the absence of meta-analytic assessment.

The purpose of this review is to analyse the effects of perceived and received social support on subjective burden among informal caregivers of an adult or older adult.

## Methods

### Design

To achieve the above objectives, we conducted a quantitative systematic review with meta-analysis. For the review, we followed the methodology proposed by Roe [26] and the reporting standards of the PRISMA Statement [27], where appropriate.

### Literature search

Electronic databases (MEDLINE—PubMed-, CINAHL—EBSCO-, EMBASE—Elsevier-, PsycINFO—ProQuest-, Scopus—Elsevier- and ISI Proceedings) were searched without time or language limits. The searching terms used across previous databases were social support, informal support, social network, perceived support, received support, burden, strain, role overload, caregiver and career. The searches ranged from the first year included in each database until September 2017. We also conducted manual searches of relevant scientific journals (nursing, psychological and medical) and reference lists in selected papers and previous reviews [3,9] for the period between January 1990 and September 2017.

### Eligibility criteria

Inclusion criteria used for selecting papers were: (a) original, quantitative studies (b) about informal caregivers of adult or older adult care-recipients (18 years old or more), (c) that related caregiver subjective burden and social support (d) yielding a correlation coefficient or another measure that could be transformed into a correlation coefficient.

To increase the validity of these eligibility criteria, the following considerations were taken into account: 1) we considered “informal caregivers” as unpaid caregivers (family members, friends, community members or volunteers) who care both in institutions and at home [28], and “adult or older adult care-recipients” as persons 18 years old or more who are dependent in at least one activity of daily living or instrumental activity of daily living; 2) caregiver subjective burden had to be measured as one-dimensional; 3) social support had to be classifiable into perceived or received social support. We consider received support when frequency of support were measured and perceived support when satisfaction (or adequacy) or availability were measured [25]. In this sense, tools that included without any differentiation both perceived and received social support (e.g. the Arizona Social Support Interview Schedule [29]) and perceived or received social support together with the social network size (e.g. the Lubben Social Network Scale [30]) were rejected. We only selected studies in which social support was measured with tools that included all types of functional dimensions (instrumental, informational and emotional), rejecting studies that included measures of specific functional dimensions.

Social support in selected papers was classified by two reviewers (RdPC and AFO) (agreement in 87% of the studies), using the definitions shown above [5,6]. We resolved any disagreement through discussion or, if required, we consulted a third review author (AJRM).

### Data extraction and synthesis

Two independent reviewers (RdPC and PAPM) extracted the population characteristics, the type of social support (perceived or received social support), and the effect estimates of each study using a standardized data extraction form. In case of disagreement, both reviewers examined the documents together following the decision rule identified in the data extraction protocol until consensus was reached.

The effect measure used to compute the combined effect was the correlation coefficient adjusted by the inverse of the variance using a random effects model. According to Cohen [31], values for effect size of 0.1–0.29, 0.3–0.49 and higher than 0.5 correspond to a small, moderate and large effect size, respectively. In repeated measured studies with no relation between time points, the first measure was chosen. When a study measured social support and subjective burden but did not relate them, correlation values were requested from the authors. Among the authors approached, two of them [32, 33] sent the solicited data.

### Quality assessment

Basing on the recommendations of Boyle [34] and Viswanathan et al.[35], we used the following criteria for assessing the methodological quality of the individual studies: (1) sampling: probabilistic sampling, (2) measures: presence of information about the measurement process, content validity and internal consistency in the target population or a similar population, and absence of information bias (3) control for confounding factors: at least one measure of objective burden must be controlled for, and (4) adequate statistical analysis.

Regarding the control of confounders, we decided to control objective burden because this construct is the most intimately related with subjective burden [24]. Objective burden comprises functional capacity, cognitive impairment and behavioural problems [36]. Because previous measures are highly correlated [18], we decide to control at least one of them. We consider that confounders are controlled when the allocation between the groups or match groups is adequate (e.g., through stratification, matching, propensity scores) or confounders are taken into account in the design and/or analysis (e.g., through matching, stratification, interaction terms, multivariate analysis, or other statistical adjustment such as instrumental variables) [35]. In case of statistical adjustment, we consider that there is no confounding bias when the variation of the point estimate is less than 10% [37].

To meet criteria, criteria 2 and 4 were considered as mandatory. Two independent reviewers (RdPC and MRM) assessed the included studies. Any disagreement was resolved by discussion or by involving a third assessor (AJRM). We chose objective primary stressors because the caregiving literature shows that these factors are the main determinants of subjective burden [18].

Following the recommendations of Meader et al. [38], based on the Grading of Recommendations Assessment, Development and Evaluation (GRADE) system [39], imprecision, inconsistency and risk of publication bias of the results of the meta-analysis were assessed. Imprecision was evaluated by the number of included studies (large: >10 studies, moderate: 5–10 studies and small: <5 studies) and the median sample size (high: >300 participants, intermediate: 100–300 participants and low: <100 participants). Inconsistency was measured through heterogeneity of findings in individual studies. Publication bias was evaluated by analysing the funnel plot and by the statistical tests explained in the Analysis section.

All reviewers participating in selection, extraction and quality evaluation of the studies were bilingual.

### Analysis

Following the recommendations of Cooper et al. [40], a random effects model was used for the meta-analysis in order to improve the generalization of the findings to any caregiver of an adult or older adult care-recipient.

The Q test was used for the analysis of heterogeneity, together with the degree of inconsistency (I^2^) of Higgins et al. [41]. Following the recommendations of Guyatt et al. [42], we used several methods for evaluating publication bias in order to strengthen the findings. These methods were evaluation of the funnel plot, the Begg’s test [43], the Egger’s test [44] and the Trim and Fill method [45]. The Begg’s test and the Egger’s test evaluate the asymmetry of the funnel plot. In these tests, a p value less than 0.10 suggests publication bias, that is, the publication or non-publication of research findings, depending on the nature and direction of the results [40]. The Trim and Fill method computes the combined effect considering a possible publication bias [45].

Sensitivity analyses were carried out to investigate the robustness of the findings. We used the leave-one-out method and subgroup analyses. The leave-one-out method consists of, given k studies, performing k-1 meta-analyses removing one study and analysing the remaining k-1 studies each time. The subgroup analyses were conducted to analyse differences between subgroups based on quality criteria or type of perceived support.

Analyses were performed using the Comprehensive Meta-Analysis 3.3 software.

## Results

**Fig 1:**
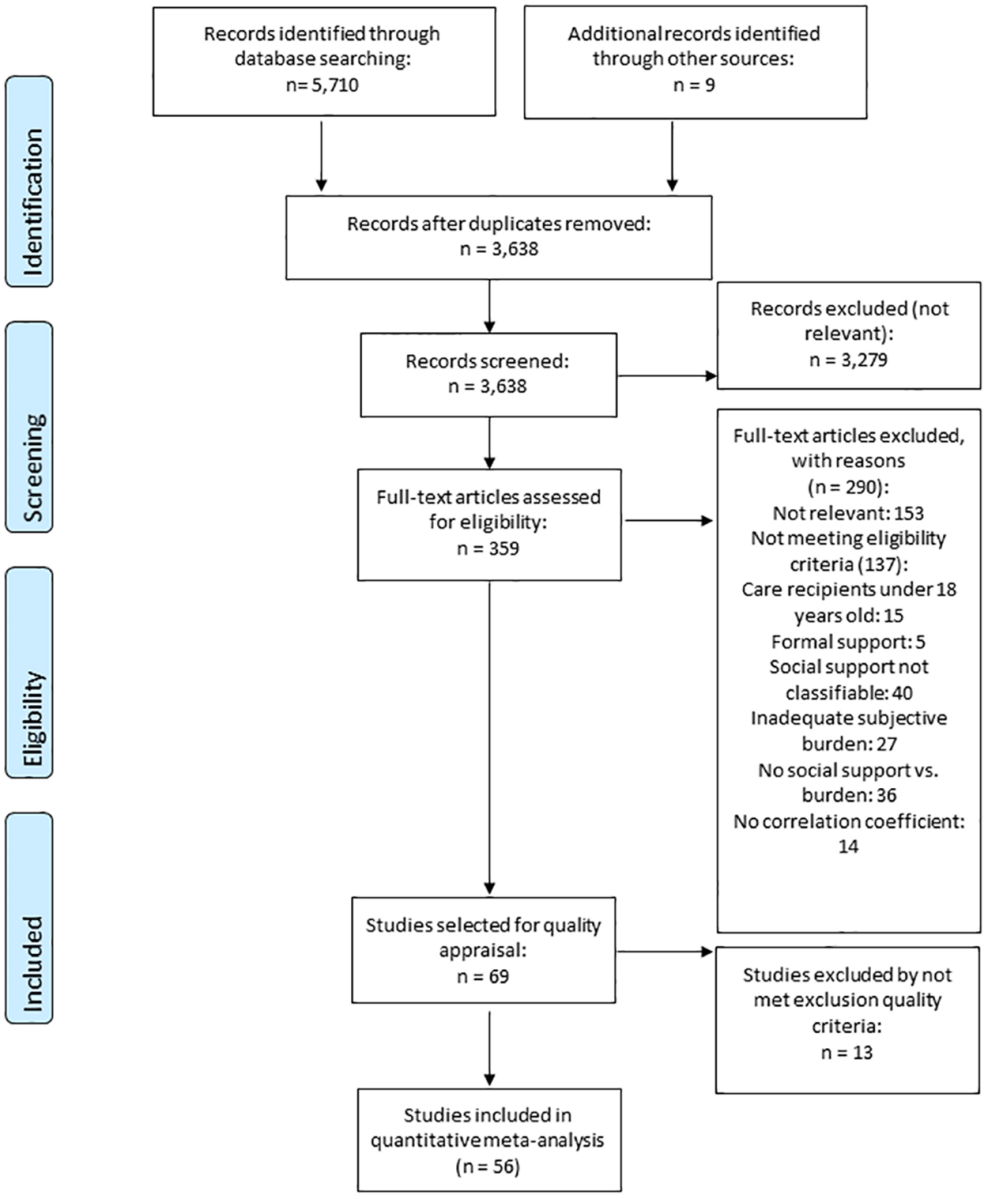
PRISMA flow diagram of the review process.

A total of 5,710 records were retrieved from the databases searched. In addition, nine references were achieved from searching the references of included articles (Fig 1). After removing duplicates, 3,638 records were screened, of which 3,279 were excluded as not being relevant. Thus, 359 documents were assessed for eligibility, of which 153 were excluded by being not relevant and 137 by not meeting the eligibility criteria. Finally, 69 documents were selected for quality assessment, of which 56 met the quality criteria 2 and 4, and were finally included in the review (40 with perceived social support [32, 33, 46–83], 13 with received social support [21, 23, 84–94] and 3 with both measures [95–97]). We analysed each type of support separately. A summary of the results is shown in Table 1.

**Table 1:** Summary of the meta-analyses’ results.

	Studies	Samples	N	Mean per sample	*r*	95% CI		I^2^	Publication bias			
						Lower	Upper		Egger’s testp-value	p-value Begg’s test	Trim and fill	
											Estimate	Variation
Perceived	43	46	6,246	135.8	-0.36	-0.40	-0.32	1.7%	0.88	0.99	-0.39	8.3
Received	16	16	7,227	451.7	-0.05	-0.095	-0.001	20.1%	0.8	0.13	-0.05	0.0

Abbreviations: *r*: combined correlation coefficient, CI: confidence interval, I^2^: degree of inconsistency.

### Perceived social support

Forty-three studies [32, 33, 46–83, 95–97] relating perceived social support and subjective burden were included in this review (Table 2). These studies contained 46 independent samples with 46 independent comparisons. Most of these studies had non-probabilistic samples (n = 41) and had no control for confounders (n = 29). The main care recipients were persons with dementia (n = 11) and frail older adults (n = 10). Regarding design, 41 were cross-sectional, one was repeated measured study with cross-sectional measures in each time point and one was case-control. Regarding the type of perceived social support, 24 studies measured satisfaction with support and 19 availability of support.

**Table 2:** Description of the studies includes for perceived social support and subjective burden.

Author, year	Care recipients	N	Sampling	Design	Type of perceived support	Control of confounders
Alvarez-Ude 2004 [46]	Dialysis	221	Non-probabilistic	Cross-sectional	Satisfaction	Yes
Anissa 2015 [47]	Mental illness	120	Non-probabilistic	Cross-sectional	Satisfaction	Yes
Artaso 2003 [48]	Dementia	80	Non-probabilistic	Cross-sectional	Satisfaction	No
Bainbridge 2009 [49]	Terminally ill	132	Non-probabilistic	Cross-sectional	Satisfaction	No
Blake 2000 [50]	Stroke	222	Non-probabilistic	Cross-sectional	Availability	No
Burton 2008 [95]	Terminally ill	50	Non-probabilistic	Cross-sectional	Availability	No
Cassidy 2013 [51]	Cancer	842	Non-probabilistic	Cross-sectional	Satisfaction	No
Cheng 2013 [96]	Dementia	142	Non-probabilistic	Cross-sectional	Availability	No
Chiou 2009 [52]	Older adults	301	Non-probabilistic	Cross-sectional	Availability	No
Clair 1995 [53]	Older adults	110	Non-probabilistic	Cross-sectional	Satisfaction	No
Davis 2009 [32]	TBI	114	Non-probabilistic	Cross-sectional	Availability	No
Del-Pino-Casado 2014 [54]	Older adults	208	Probabilistic	Cross-sectional	Satisfaction	Yes
Edwards 2002 [55]	Parkinson	41	Non-probabilistic	Cross-sectional	Satisfaction	No
Folkman 1994 [97]	HIV	82	Non-probabilistic	Cross-sectional	Satisfaction	No
Gallart 2013 [56]	Older adults	110	Non-probabilistic	Case-control	Satisfaction	No
Goldsworthy 2008 [33]	Parkinson	136	Non-probabilistic	Cross-sectional	Satisfaction	Yes
Goris 2016 [57]	COPD	112	Non-probabilistic	Cross-sectional	Availability	Yes
Greenberger 2003 [58]	Older adults	240	Probabilistic	Cross-sectional	Satisfaction	No
Hanks 2007 [59]	TBI	60	Non-probabilistic	Cross-sectional	Availability	Yes
Kahriman 2015 [60]	Cancer	80	Non-probabilistic	Cross-sectional	Availability	No
Kaur 2014 [61]	Mental illness	100	Non-probabilistic	Cross-sectional	Availability	No
Leibach 2013 [62]	Multiple sclerosis	81	Non-probabilistic	Cross-sectional	Availability	No
Liu 2012 [63]	Dementia	96	Non-probabilistic	Cross-sectional	Availability	Yes
Lopez Alonso 2005 [64]	Older adults	215	Non-probabilistic	Cross-sectional	Satisfaction	No
Losada 2010 [65]	Dementia	468	Non-probabilistic	Cross-sectional	Satisfaction	Yes
Majerovitz 2007 [66]	Older adults	103	Non-probabilistic	Cross-sectional	Satisfaction	Yes
Manso-Martínez 2013 [67]	Older adults	88	Non-probabilistic	Cross-sectional	Satisfaction	Yes
Marwit 2002 [68]	Dementia	166	Non-probabilistic	Cross-sectional	Satisfaction	No
Molina Linde 2005 [69]	Dementia	46	Non-probabilistic	Cross-sectional	Satisfaction	No
Möller-Leimkühler 2012 [70]	Mental illness	102	Non-probabilistic	Cross-sectional	Availability	No
Moral 2003 [71]	Older adults	215	Non-probabilistic	Cross-sectional	Satisfaction	No
Muñoz Bermejo 2015 [72]	Older adults	107	Non-probabilistic	Cross-sectional	Satisfaction	No
Pushkar Gold 1995 [73]	Dementia	118	Non-probabilistic	Repeated measures	Availability	Yes
Reis 1994 [74]	Dementia	213	Non-probabilistic	Cross-sectional	Availability	Yes
Ryan 2012 [75]	Dementia and Older adults	135	Non-probabilistic	Cross-sectional	Availability	No
Shieh 2012 [76]	Cancer	100	Non-probabilistic	Cross-sectional	Availability	No
Son 2003 [77]	Dementia	107	Non-probabilistic	Cross-sectional	Satisfaction	Yes
Stevens 2013 [78]	TBI	90	Non-probabilistic	Cross-sectional	Availability	No
Teixeira 2012 [79]	Cancer	214	Non-probabilistic	Cross-sectional	Satisfaction	Yes
Verez Cotelo 2015 [80]	Dementia	25	Non-probabilistic	Cross-sectional	Satisfaction	No
Wang 2011 [81]	Terminally ill	178	Non-probabilistic	Cross-sectional	Satisfaction	No
Yüksel 2013[82]	Mental illness	103	Non-probabilistic	Cross-sectional	Availability	No
Yurtsever 2013 [83]	Dementia	107	Non-probabilistic	Cross-sectional	Availability	No

Abbreviations: TBI. Traumatic brain injury, HIV: human immunodeficiency virus, COPD: Chronic Obstructive Pulmonary Disease.

**Fig 2:**
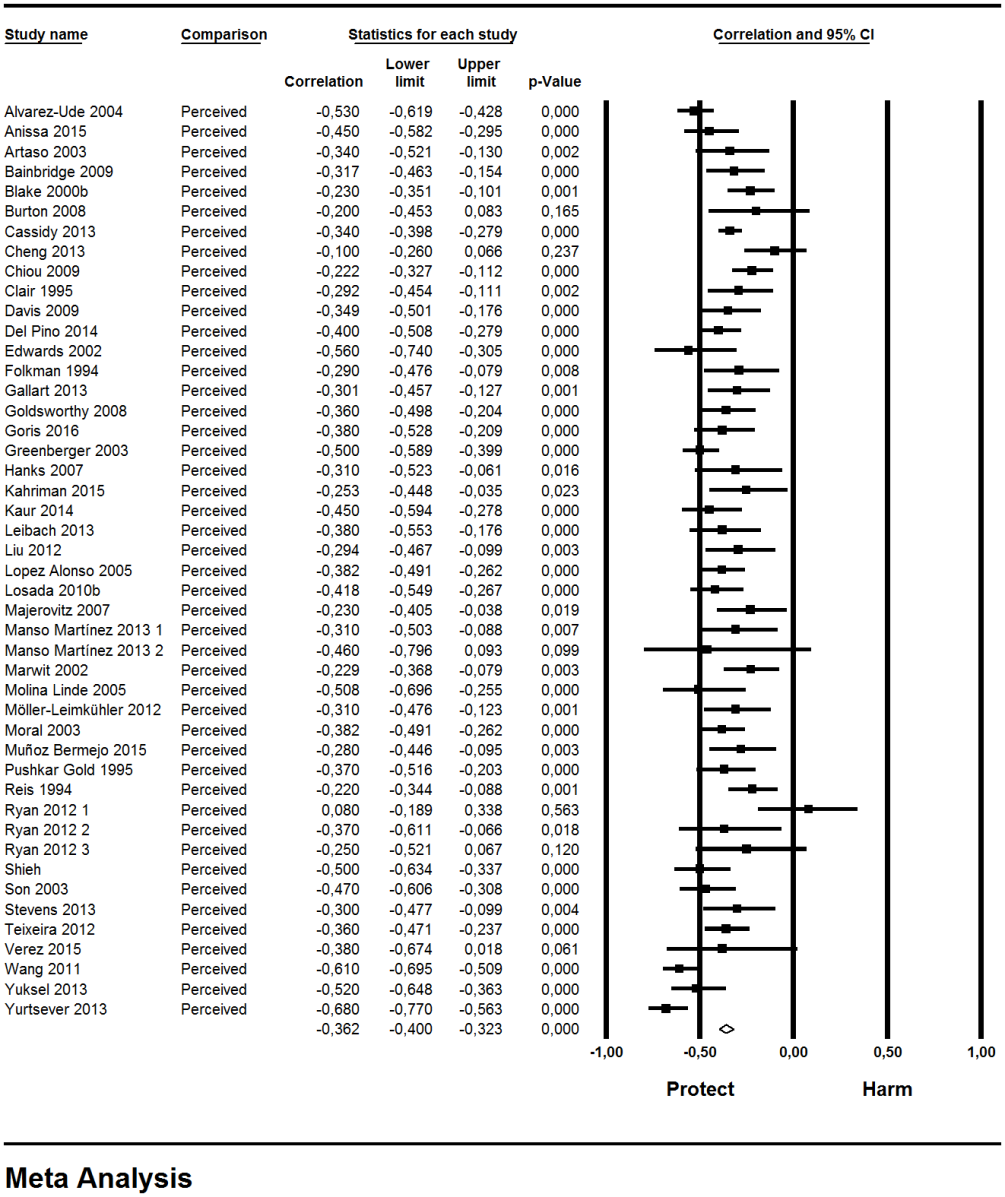
Forest plot for perceived social support and subjective burden.

The combined effect (*r* = -0.36; 95% CI = -0.40, -0.32; N = 6,246; median sample size: 135.8) showed a moderate, negative effect (Table 1). Thus, caregivers with high perceived support experience less subjective burden. The effects in the individual samples were negative (except in one) and statistically significant (except in six studies) (Fig 2). The leave-one-out method yielded variations in the combined estimate under 2.2% (from -0.353 to -0.368). We consider the result of the meta-analysis as precise because of the width of the confidence intervals, the numbers of studies included and the median sample size. A very low heterogeneity was found among the results of these individual studies (Q = 45.8, degree of freedom [df] = 45, p> 0.10, I^2^ = 1.7%).

**Fig 3:**
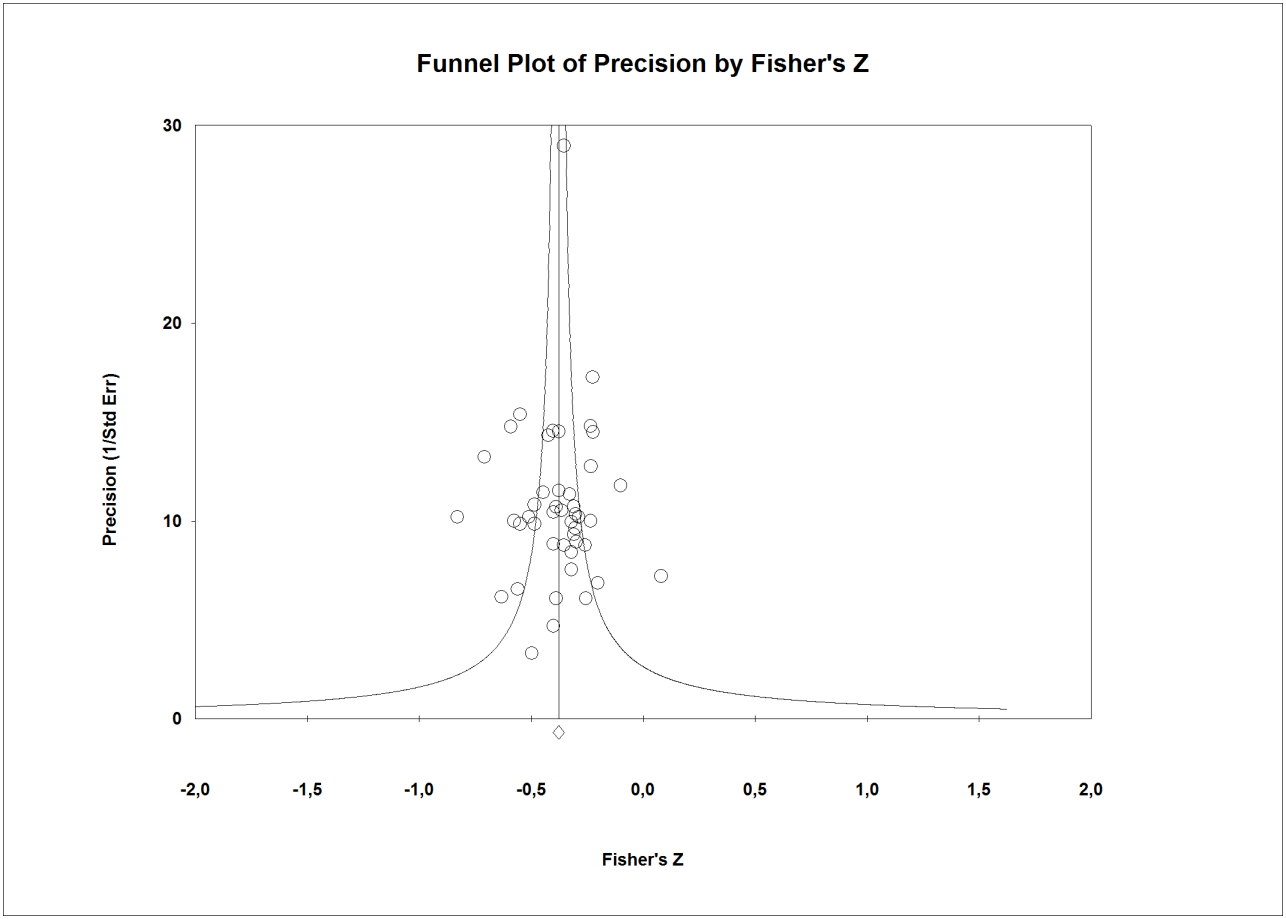
Funnel plot for perceived social support and subjective burden.

Regarding publication bias, the funnel plot (Fig 3) seems somewhat symmetric. The small studies did not show any tendency regarding their effect sizes and these studies had no bigger effect sizes than larger studies. The Egger’s test (p = 0.88) and the Begg’s test (p = 0.99) excluded the publication bias. In addition, the combined effect calculated by the Trim and Fill method (*r* = -0.39) varied by 8.3%.

No differences were found between studies controlling objective primary stressors (*r* = -0.37; 95% CI = -0.42, -0.32; 14 samples) and those not controlling it (*r* = -0.36; 95% CI = -0.41, -0.31; 32 samples).

Regarding the type of perceived social support, no differences were found between studies measuring satisfaction with social support (*r* = -0.39; 95% CI = -0.43, -0.35; 25 samples) and those measuring availability of social support (*r* = -0.33; 95% CI = -0.40, -0.26; 21 samples).

### Received social support

Sixteen studies [21, 23, 84–97] relating received social support and subjective burden were incorporated in the present review (Table 3). These studies included 16 independent samples with 16 independent comparisons. These studies were cross-sectional (n = 14) or repeated measured studies with cross-sectional measures in each time point. All the studies had non-probabilistic samples and most of these studies (n = 12) had no control for confounders. The main care recipients were frail older adults (n = 5).

**Table 3:** Description of the studies includes for received social support and subjective burden.

Author, year	Care recipients	N	Sampling	Design	Control of confounders
Adriansen 2011 [84]	Stroke	180	Non-probabilistic	Repeated measures	No
Burton 2008 [95]	Terminally ill	50	Non-probabilistic	Cross-sectional	Yes
Cheng 2013 [96]	Dementia	142	Non-probabilistic	Cross-sectional	No
Dorfman 1996 [85]	Older adults	80	Non-probabilistic	Cross-sectional	No
Folkman 1994 [97]	HIV	82	Non-probabilistic	Cross-sectional	No
Greene 2013 [86]	HIV	96	Non-probabilistic	Cross-sectional	No
Kruithof 2016 [23]	Stroke	183	Non-probabilistic	Repeated measures	No
Losada 2010 [87]	Dementia	334	Non-probabilistic	Cross-sectional	No
Meiland 2001 [88]	Dementia	93	Non-probabilistic	Cross-sectional	No
Riemsma 1999 [89]	Rheumatoid arthritis	174	Non-probabilistic	Cross-sectional	No
Robinson 1990 [90]	Mentally impaired	78	Non-probabilistic	Cross-sectional	No
Rodakowski 2012 [91]	Spinal cord injury	173	Non-probabilistic	Cross-sectional	Yes
Spaid 1994 [92]	Older adults	131	Non-probabilistic	Cross-sectional	No
Stommel 1990 [93]	Older adults	307	Non-probabilistic	Cross-sectional	Yes
Tang 2006 [94]	Older adults	325	Non-probabilistic	Cross-sectional	Yes
Verbakel 2016 [21]	Older adults	3,986	Non-probabilistic	Cross-sectional	No

Abbreviations: HIV: human immunodeficiency virus.

**Fig 4:**
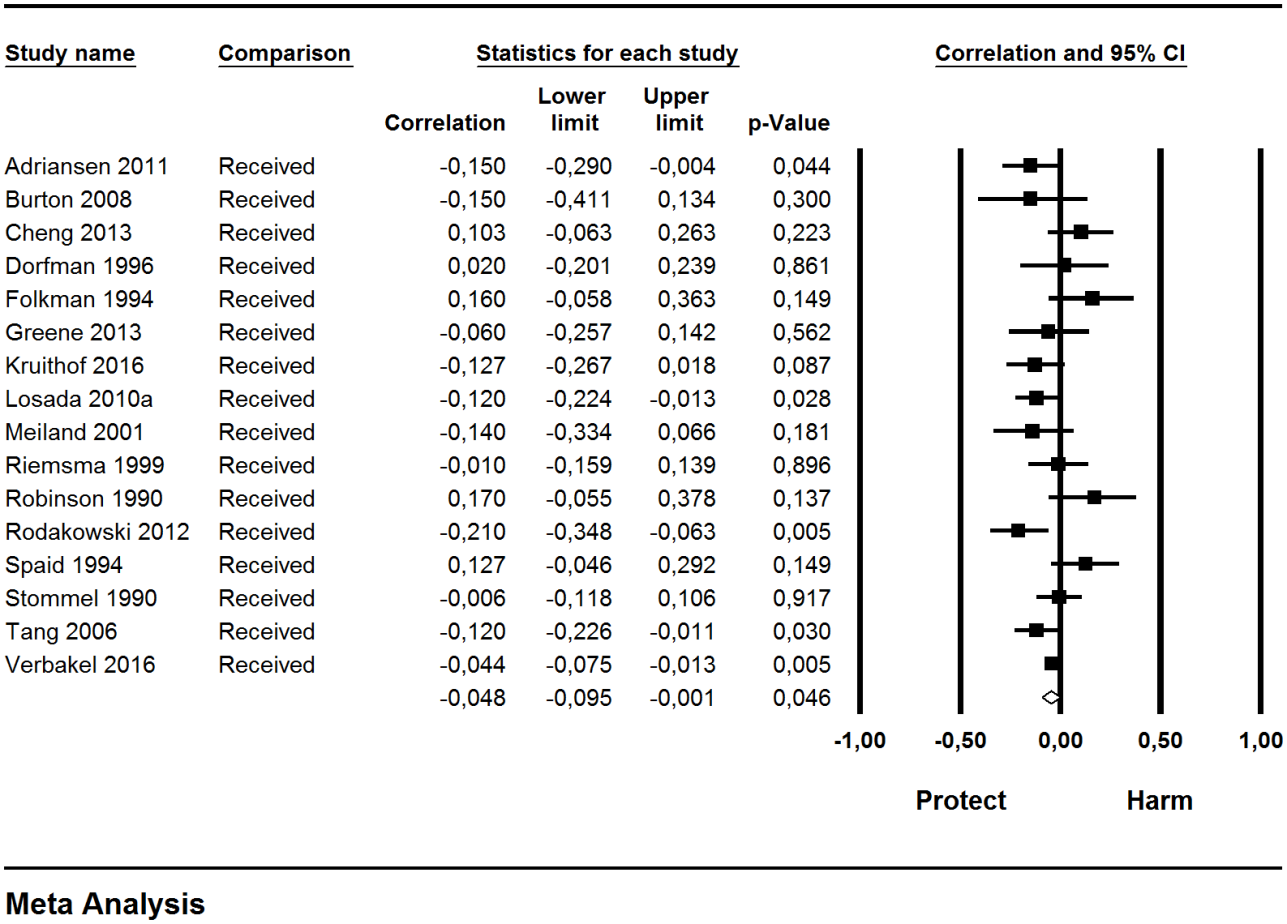
Forest plot for received support and subjective burden.

The combined effect (*r* = -0.05; 95% CI = -0.095, -0.001; N = 7,227; median sample size: 451.7) showed a very small, negative effect (Table 1). Thus, caregivers with high received support experience less subjective burden but the effect size of this relationship is very small. The effects in the individual studies were negative in 9 cases and positive in 7, and statistically significant in 5 cases (Fig 4). The leave-one-out method yielded variations in the combined estimate from 15.5% (*r* = 0.038) to 28.8% (*r* = 0.058), and the result of the meta-analyses turned out to be non-statistically significant in 10 cases. We consider the result of the meta-analysis as precise because of the width of the confidence intervals, the numbers of studies included and the median sample size. A low to moderate heterogeneity was found among the results of the individual studies (Q = 18.78, df = 15, p> 0.10, I^2^ = 20.14%).

**Fig 5:**
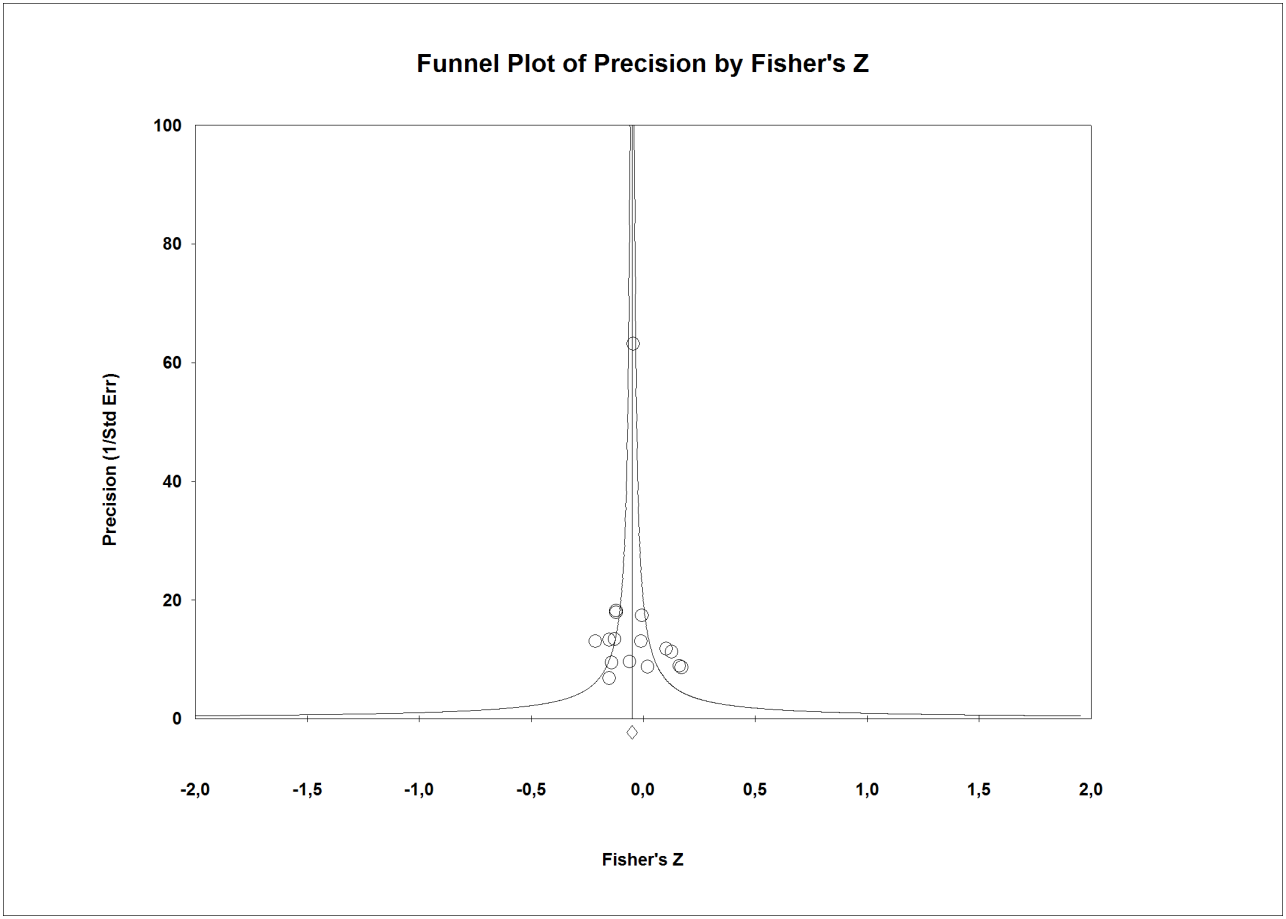
Funnel plot for received social support and subjective burden.

Concerning publication bias, the funnel plot (Fig 5) seems somewhat symmetric. The small studies did not show any tendency regarding their effect sizes and these studies had no bigger effect sizes than larger studies. The Egger’s test (p = 0.80) and the Begg’s test (p = 0.13) excluded publication bias. Furthermore, the combined effect calculated by the Trim and Fill method (*r* = -0.05) did not vary.

No differences were founds between studies controlling objective primary stressors (*r* = -0.07; 95% CI = -0.16, 0.01; three studies) and those not controlling it (*r* = -0.04; 95% CI = -0.09, 0.02; 13 studies).

## Discussion

In the present review, subjective burden has been negatively related to perceived social support (with a moderate effect size) and received social support (with a very low effect size), in caregivers of an adult or older adult care-recipient.

The findings of the meta-analyses performed were reasonably accurate, valid and robust, based on the high number of included studies, the low level of heterogeneity of the results in individual studies, the low risk of publication bias, and the sensitivity analysis. Regarding the methodological quality of the included studies, there is a low risk of classification bias and errors in analysis in these studies, but most of the included studies had non-probabilistic samples and did not control for confounders. One of the main problems in systematic reviews of observational studies is the control of confounders [35]. In this review, we included strategies for evaluating this issue and demonstrated that the control of objective burden in individual studies did not affect the results of the present meta-analysis. On the other hand, all the studies included in this review, except one, were cross-sectional or repeated measures studies that did not relate to the different time points. So, no causal relationship can be extracted from the present review. However, the findings in the present study are consistent with a caregiving theoretical framework in which social support is related to subjective burden [21], although no causation sequence can be established.

The present review showed that the relationships between social support and subjective burden depend on whether the social support is measured as perceived or received. This issue can explain the mixed results commented upon in the background section [9,20]. Our findings contribute to explain these mixed results, demonstrating that the relationships between social support and subjective burden can vary according the type of support measured.

The different relationship between received and perceived support with subjective burden could be due to variations of measures of social support, context, type of caregivers, or confounders. Because we only selected studies in which social support included all types of functional dimensions, it is unlikely that variations between perceived and received support regarding subjective burden are due to variations in the type of functional dimension. The low heterogeneity found in the meta-analyses support previous idea and underline that the above-mentioned differences between received and perceived support are not due to context or type of caregivers. Last, because we have demonstrated that studies with adequate control of confounders are similar results to those that do not.

In this review, perceived social support is more consistently related to subjective burden than received social support. In addition, the relationship of perceived social support with subjective burden has a bigger effect size than that of received social support. These findings are consistent with those of studies in other populations [25,98], in which perceived support is more strongly related with health indicators than received support. Our findings support the consideration of perceived social support as a possible good predictor of subjective burden in caregivers of adult or older adult care-recipients.

Since subjective burden is the consequence of the evaluation of stressors in the caregiving situation [99], the negative relationship between this construct and perceived social support could prove that the perception of the social support as adequate is related to appraising a situation as less stressful, according with the approach of Cohen et al. [12,13]. However, our findings do not discard the inverse hypothesis (subjective burden can lead to a worse evaluation of social support) or the reciprocal influence.

Regarding received social support and subjective burden, our findings extend the current knowledge showing the relationship between them as clinically irrelevant in caregivers. Therefore, the theoretically argued [12,13] stress-buffer effect of received social support is scarce. This clinical irrelevance could be explained by the findings of Melrose et al. [25], which showed that received support is related to emotional health when the need for support was considered, that is, when received support was measured as the number of times support was received when needed. In contrast, received support is not related to emotional health when it was measured as the number of times support was received [25].

Thus, our findings extend the evidence that perceived and received support are not redundant constructs. Therefore, models, research and clinical questions must take into account their separable and joint influence.

Several studies have analysed the efficacy of social support interventions in caregivers, yielding heterogeneous outcomes [100]. When intervening on social support, two targets are possible [101]: 1) closer family members, increased frequency of seeing others and/or more emotional support (promoting perceived social support through increasing “feeling connected”) and 2) validation and building of new friendships (promoting received social support through “building connections”). Our findings broadly support interventions to promote social support in caregivers to prevent or alleviate subjective burden, and specifically, to intervene on “feeling connected” more than on “building connections” when preventing or alleviating burden.

Therefore, our findings support the use of perceived rather than received social support when preventing or alleviating subjective burden, because perceived support may be better predictor of subjective burden than received support and interventions to promote perceived support to reduce subjective burden may be more appropriate than those promoting received support.

As we discussed above, our study had the limitation that all the studies included in this review, except one, were cross-sectional or repeated measures studies that did not relate to the different time points (no causal relationship can be extracted).

## Conclusions

Several conclusions can be extracted from this study, regarding caregivers of adult and older adult care-recipients: 1) perceived and received support are not redundant constructs, 2) the relationships between social support and subjective burden depend on whether the social support is measured as perceived or received, 3) the relationship of perceived social support with subjective burden has a bigger effect size than that of received social support, the relation between received support and subjective burden being clinically irrelevant, 4) perceived social support may be a good predictor of subjective burden, 5) the perception of social support as adequate may be related to appraising a situation as less stressful, 6) Our findings broadly support interventions to promote social support in caregivers to prevent or alleviate subjective burden, and specifically, to intervene on the promotion of perceived social support more than on the promotion of received social support when preventing or alleviating burden.

For future research, more longitudinal studies are needed to enhance the causal relationships between social support and subjective burden.

## Supporting information

S1 Checklist PRISMA checklist for systematic reviews. (DOCX)
